# Sacredness of the Medical Profession

**Published:** 1881-05

**Authors:** W. R. Monroe

**Affiliations:** Baltimore


ARTICLE III.

### SACREDNESS OF THE MEDICAL PROFESSION.

BY W. R. MONROE, M. D., BALTIMORE.

In the family the mission of the physician is more intimate, sacred, and confidential than that of the pastor. The latter is supposed to know the family affairs in a general way, as to their circumstances morally, relatively and in a worldly point of view, hence the pastor addresses himself to the general health of his parishioners, their worldly prospects, and more especially to their spiritual needs. His counsels are wise, his sympathies strong, and his prayers are fervently enlisted for both their spiritual and temporal welfare, consequently his services are valued and most highly prized by all the members of the household.

But the family physician is expected to be informed as to the details, the habits and manner of living among every member of the family. Physical ailments are traceable to a great variety of causes, including mental and social troubles of every kind; private habits and the secrets of individual life must be revealed to the family doctor. He must be informed concerning home life as well as out door life. If there is trouble between housekeeper and domestic, man and wife, or parent and child, it behooves the physician to know it, as domestic troubles may effect the health of individuals. Mistakes, disappointments and disabilities, both mental and physical, are essential elements in forming a correct diagnosis in human diseases. And it is expected that a family physician will sacredly keep all the secrets of a household committed to him, and give wise and good counsels, as well as write prescriptions; how important, therefore, that he, himself, should be a good man, and a true man!

How then can vain and pompous young graduates, or skeptical practitioners, or profligate and profane doctors of medicine, expect to obtain the patronage of Christian families, or to retain the favor and good will of respectable and morally intelligent people? They ought not. For a pastor to be found indulging in bad conduct would be to dismiss him from the confidence of every family as a spiritual adviser; why then should an avowed sinner in the medical profession be entrusted with the secrets of individual, or family affairs, and be taken into their confidence? Is such a man, however well educated in medicine, competent to administer the needed consolation in a heart-stricken household?

It is not contemplated, or implied, that a good-hearted doctor shall carry a solemn countenance, or a long face into the sick room; but on the contrary, every good man should be always cheerful, and every righteous physician will enter the chamber of his patient with a cheerful and genial spirit; his countenance will beam with sympathy, good cheer and hopefulness for his patient. The good physician who always carries the sunshine of love into the sick chamber, largely contributes by that means to the welfare of his patient. To do good and be useful, one must be good at heart. "Make the tree good and his fruit will be good also," is an established truth. Oh! that all physicians would consider the sacredness of their profession, and become men of clean hands and pure hearts as well as of studious habits, and then, as one has wisely expressed himself, we shall "gaze into a hopeful future and be filled with glowing and bright pictures of the era when medical science shall be

"Above the reach of sacrilegious hands,
Whose honors with increase of ages grow,
As streams well down, enlarging as they flow."

